# Inspection of Biomimicry Approaches as an Alternative to Address Climate-Related Energy Building Challenges: A Framework for Application in Panama

**DOI:** 10.3390/biomimetics5030040

**Published:** 2020-08-24

**Authors:** Miguel Chen Austin, Dagmar Garzola, Nicole Delgado, José Ulises Jiménez, Dafni Mora

**Affiliations:** 1 Research Group in Energy and Comfort in Bioclimatic Buildings (ECEB), Faculty of Mechanical Engineering, Universidad Tecnológica de Panama, Panama City 0801, Panama; miguel.chen@utp.ac.pa (M.C.A.); dagmar.garzola@utp.ac.pa (D.G.); nicole.delgado@utp.ac.pa (N.D.); ulises.jimenez@utp.ac.pa (J.U.J.); 2 Centro de Investigaciones Hidráulicas e Hidrotécnicas (CIHH), Panama City 0801, Panama; 3 Centro de Estudios Multidisciplinarios en Ciencias, Ingeniería y Tecnología (CEMCIT-AIP), Panama City 0801, Panama

**Keywords:** biomimicry, buildings, energy efficiency, energy regulations, SWOT analysis

## Abstract

In the Panama context, energy consumption in the building sector is mostly related to the conditioning of indoor spaces for cooling and lighting. Different nature strategies can be mimic to strongly impact these two aspects in the building sector, such as the ones presented here. A comprehensive analysis regarding literature related to biomimicry-based approaches destined to improve buildings designs is presented here. This analysis is driven by the increasing energy regulations demands to meet future local goals and to propose a framework for applications in Panama. Such biomimicry-based approaches have been further analyzed and evaluated to propose the incorporation of organism-based design for three of the most climate types found in Panama. Consequently, a SWOT analysis helped realized the potential that biomimicry-based approaches might have in improving the odds of in meeting the local and global regulations demands. The need for multidisciplinary collaboration to accomplish biomimicry-based-designed buildings, brings an increment in the competitivity regarding more trained human-assets, widening the standard-construction-sector thinking. Finally, the analysis presented here can serve as the foundation for further technical assessment, via numerical and experimental means.

## 1. Introduction

Worldwide organizations such as the European Union (EU), the International Energy Agency (IEA) and the International Renewable Energy Agency (IRENA) have invested considerable effort in studying energy alternatives to reduce global energy consumption from fossil fuels and to promote investment in renewable energy, along with more rational consumption by society [1]. Due to the well-known current situation of fossil fuels and the evidence of global warming, several energy policies have been established. They have decided to abruptly reduce the use of internal combustion vehicles, non-renewable thermoelectric plants, and encourage the development of electric vehicles, wind, and solar generation.

In recent years, biomimicry has become relevant in engineering and architecture, giving way to intelligent, self-sustaining, and curious buildings in terms of shape and structure. The energy usage in buildings and the production of construction materials is a significant part of the total energy consumption in today’s societies. We are currently facing two main problems related to energy consumption: the depletion of energy resources and mineral raw material, and the increase in various types of pollution causing global warming [2].

From the first-ever humans, humankind has been inspired by nature, imitating nature consciously, or subconsciously. By examining the anatomy and flying techniques of birds, Leonardo Da Vinci was a biomimicry pioneer, which inspired the Da Vinci Index. This index aimed to increase the effectiveness of biomimicry applications [3]. Many of these innovations are renowned in areas of medical and pharmaceutical sciences; shelter architectures; weapons and defense, including armors, sensors, and alarm systems; agriculture and food production; and processes related to manufacturing [4].

Biomimicry is defined as the art of imitating nature, a thing that we have been doing since a long time ago, trying to solve problems of food, shelter, and more, taking as a source of inspiration our natural surroundings [5]. Biomimicry is more than being inspired by nature; rather than creating something sustainable, it is to understand how it survives and evolves around us being self-sustainable. Nature has had to be creative to sustain life. It has produced models, patterns, and solutions that have been selected and tested over time. In these solutions, many of the problems that we face today are solved [6].

**Figure 1:**
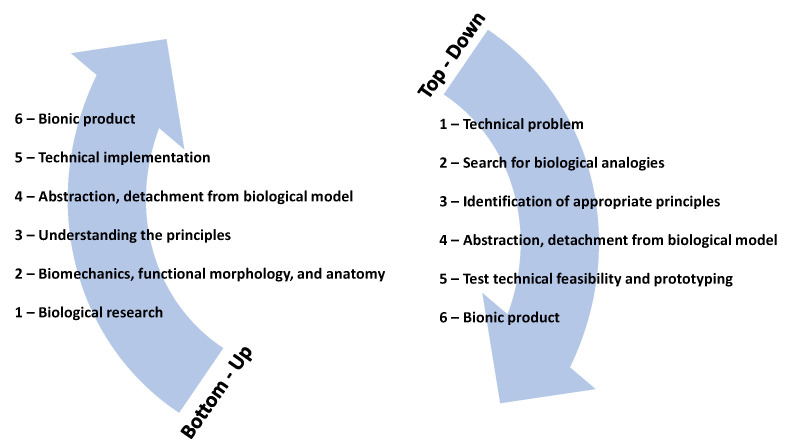
Biomimicry approaches (adapted from [7]).

Looking for strategies, designers have adopted the knowledge of biological research. This knowledge influences human designs, rather than first determining human design problems. This approach is called “bottom-up approach” and “solution-driven biologically inspired design” (Figure 1) [7]. Principal concerns in such biomimetic strategies chosen are the extent of possibilities, the challenges in representing the biophysical knowledge, and the difficulties encountered in the abstraction of relevant principles [8].

With the ultimate purpose of discovering those hidden solutions that nature keeps, the interest in biomimicry research has increased, mainly in the field of architecture and engineering since it offers new and inspiring solutions while creating the possibility of sustainability in the built environment. Some of these solutions are currently being used, although due to aesthetics or functionality, they are not visually appreciated or not evident. Some other solutions are notorious and can be found in large cities or remote areas. In contrast, other solutions may exist only in the mind of an engineer or an architect, waiting to be executed, and many others are still waiting to be discovered [6].

Researchers have introduced two main approaches to the biomimetic design process: the problem-based approach and the solution-based approach. The former is driven by inspiration from biology through a non-linear or dynamic progression, which provides feedback and refinement in the loops. On the other hand, designers seek solutions by identifying the problem. This leads biologists to relate the problem to an organism that has solved a similar problem. The solution-based approach is used when the design process initially depends on the scientific knowledge of biologists and scientists, rather than on human design problems [6,9]. Besides, the transformation of the strategies available in nature into technical solutions for biomimetic designs could become a complicated multidisciplinary process. Process complexity increases when conflicts arise by integrating a series of strategies from different organisms to attain improved solutions [8].

In [10], a framework applicable to both approaches (design looking to biology, and biology influencing design), is described. The first part of the framework determines which aspect of ‘bio’ has been ‘mimicked’ (referred to as a level). From this, three levels of mimicry were identified: the organism, behavior, and ecosystem. The organism level refers to a specific organism like a plant or animal and may involve mimicking part of or the whole organism. The second level refers to mimicking behavior and may include translating an aspect of how an organism behaves or relates to a broader context. The third level is the mimicking of whole ecosystems and the common principles that allow them to function successfully. Within each of these levels, a further five possible dimensions to the mimicry exist. The design may be biomimetic, for example, in terms of what it looks like (form), what it is made out of (material), how it is made (construction), how it works (process), or what it is able to do (function). The differences between each kind of biomimicry are described in Table 1 and are exemplified by looking at how different aspects of a termite, or ecosystem a termite is part of, could be mimicked.

Similarly, in 2015, a proposed methodology following the problem-based approach is well-defined and detailed in [8]. This methodology consists of eight steps, beginning with defining the design challenge and finishing with the performance estimation of the proposed design concept. An additional step is included to create an exploration model (a broadly used method to model systems with significant uncertainty [11,12]) for the main challenge studied: water regulation in building envelopes. Such steps are named as follows: (i) creating an exploration model for water regulation, consisting of a large database organized in hierarchical levels depending on the design challenge chosen. (ii) Defining the design challenge. (iii) Exploring possible scenarios and identifying exemplary pinnacles, based on several scenarios provided by the exploration model, which lead to different relevant pinnacles. (iv) Analysis of selected pinnacles, in terms of performance strategies, mechanism, principles, and main features, to provide a practical guideline for the design process. (v) Deriving “imaginary pinnacles,” which refers to the selection of a synthetic pinnacle (or abstraction of various pinnacles) representing a collection of dominant features for a specific function. The authors introduced this step to reduce complexity, given the high number of pinnacles to be analyzed, and proposed to employ the convergent evolution theory to facilitate categorization. This theory refers to the process where non-monophyletic organisms independently evolve similar traits, given the need to adapt to similar environmental conditions [13]. (vi) Outlining the design concept for further abstraction level is crucial to indicate successful aspects to be implemented into the design concept based on the pinnacle analysis. The authors proposed the use of a design path matrix for this step. (vii) Generating a preliminary design concept, in which the transformation of steps (iv) and (vii) is performed. (viii) Estimating performance. The building envelope performance was estimated by calculating the number of days per year, in which the envelope provides an indoor comfort level of humidity [8].

More recently, last year, a proposed methodology following the solution-based approach is well-defined and presented in [9]. This methodology is described for three major steps: the biological domain which manages the input data gather from the extraction of pinnacles’ strategies, the transfer phase where analysis of biological solutions can target current building-related problems and challenges, and finally, the technological domain where the biological solutions identified are classified on how they can be used in buildings with both objective and application level (i.e., envelope, component, system, structure).

The case studied lies in biomimicry strategies presented in cold-blooded animals (or ectotherms), which can be compared to nearly zero energy buildings (nZEB): as ectotherms require less food to meet biological needs and make use of external energy sources, nZE-based designed building intent to ensure indoor thermal comfort (as biological needs for humans) while lowering the energy consumption, and also, promoting the use of local external energy sources like from photovoltaic solar panels.

### 1.1. Motivation and Objective

Although it has large hydroelectric projects installed, Panama has relied on fossil fuels to meet national electricity demand [14,15], which varies depending on environmental conditions. Throughout the year, the energy demand can be supplied with 80% renewable energy. However, in the dry season, when the dams decrease their levels, the participation of fossil fuels can be found above two-thirds of the total. Energy policies such as laws 45, 37, and 44 strongly encourage micro-hydroelectric plants, photovoltaics, and wind farms [16].

In this matter, Panama’s national energy policy considers the following actions for the sustainable development of the energy sector [17]: (i) elaboration of a long-term plan, (ii) comprehensive management of watersheds, (iii) territorial ordering, (iv) allocation of a price to the carbon content of energy, (v) implementation of a law for the rational and efficient use of energy, (vi) reorganization of the laws of renewable sources, sustainable cities, energy, and education program, (vii) and electric mobility.

The National Energy Plan 2015–2050 (PEN in Spanish) consists of regulations for rational and efficient use of energy are established, as well as educational programs in which efforts are made to build with more environmentally and energy-appropriate enclosures or to modify existing buildings for efficiency. Such regulations and programs provide the population with recommendations regarding appropriate households usage, air conditioning equipment, and raise awareness of the impact of the misuse of these on the national production [18]. Accounting for the aforementioned, our investigation focuses on performing a critical literature review of different biomimicry strategies applied in buildings, searching to improve energy efficiency, and finally, to establish a framework (or baseline) for introducing their applications in Panama.

Considering the search for tools to increase the regenerative capacity of the built environment, a framework for understanding the application of biomimicry may redefine and clarify the potential of biomimicry-based actions. Such a framework may allow designers, who wish to employ biomimicry as a methodology for improving the sustainability of the built environment, to identify effective approaches [10].

### 1.2. Scope and Document Structure

**Figure 2:**
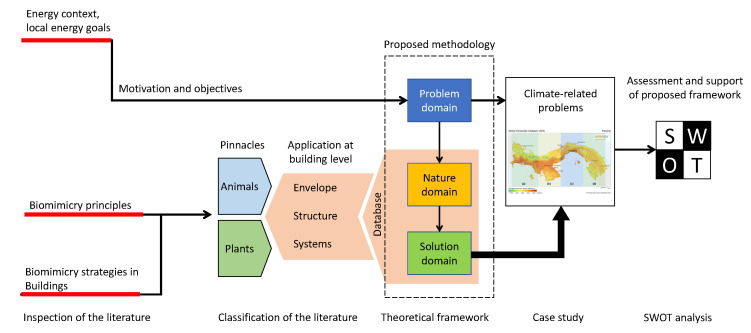
Schematic representing the structure of the paper.

The core of this study reaches building applications using strategies inspired by nature. These building applications are climate-related solution problems, and thus, the searching for solutions regarding three important climate types is of high interest: coastal areas, arid areas, and tropical forest areas. Such searching is motivated by the energy context and local energy goals established by current regulations in Panama. Therefore, this document is organized as presented in Figure 2, which is divided into three parts. The starting point lies in the theoretical framework of biomimicry strategies, followed by the motivations and objectives that are raised from the interest in meeting the current regulation objectives. This might allow us to propose a framework for introducing biomimicry-level-based strategies for building designs in Panama. Finally, possible risks are assessed by analyzing related weaknesses and threats that may arise from the introduction of such strategies in a developing country as Panama. However, also, associated strengths and opportunities are presented in a SWOT analysis to support such a framework.

## 2. Inspection of Biomimicry Approaches in Buildings: Basis for the Theoretical Framework

From the most common (green areas for aesthetics and reducing heat in buildings) to the most complex (glass panels that absorb heat from the sun and at the same time allow observing through it) are some of the examples seen from biomimetics in real life.

When an interior designer says that design is influenced by nature, it refers to its appearance: it has an organic shape. Nature is a good teacher in this regard, but imitating or being inspired by natural-looking forms, textures, and colors alone is not biomimetics. To quote Dr. Julian Vincent, “biomimetics has to have some biology in it.” By which he means that design should, in some way, be informed by nature’s science, not just its look to be genuinely biomimetic [6]. Sustainable development is moving to a new level where buildings are integral to nature, supporting nature’s work rather than interfering with life-sustaining ecosystems. Nature has been offering immense ideas and inspirations to designers for creating architecture [19].

It is essential to recognize that animals are not the only ones who adapt to these temperatures; there are many plants that have developed adaptational mechanisms. For example, rotating leaves enable the plant to orient its leave away from maximum exposure to the sun [20]. These methods aim at avoiding direct sunlight, dissipating overheating, or providing the exterior skin with good thermal insulation. These methods work as a part of a thermoregulation process to control the internal body temperature. Some plants, such as mangroves found in south Sinai, avoid direct sunlight by rotating their leaves away from sunlight, while some animals, such as lizards, rest in burrows and shaded areas or move rabidly or rise their bodies away from heated ground.

**Figure 3:**
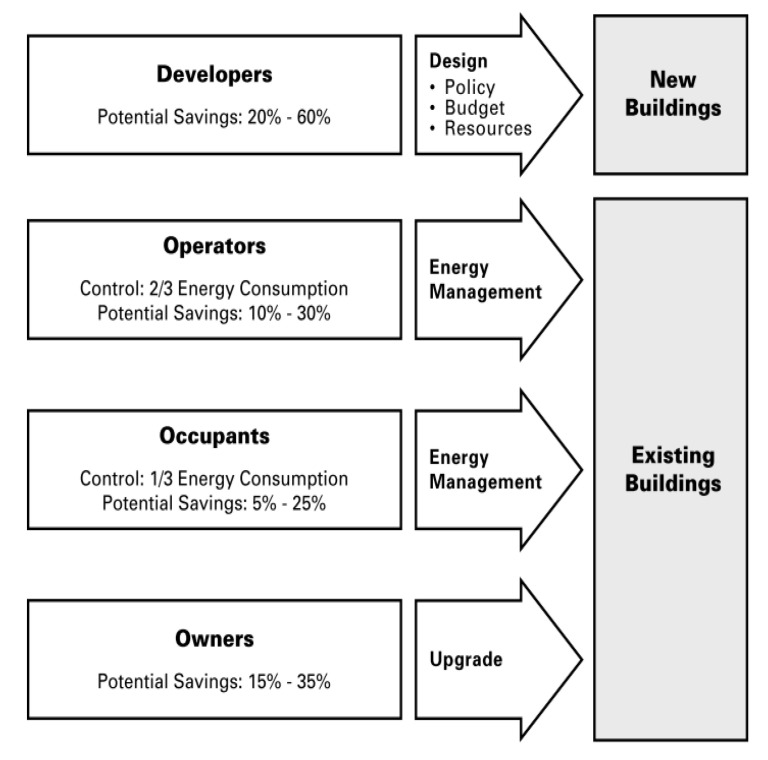
Key influences on building energy consumption [21].

The same attitude was mimicked or could be imitated in buildings as active systems. Buildings could rotate, use movable shading devices, windows, and controlled wind catch. It could also be transformed into constant features like constructing underground buildings or raise them above the heated ground with high columns [20]. Notwithstanding their different perspectives, developers who mandate energy-efficient designs, building operators who employ effective management practices, and educated, energy-conscious tenants, can significantly reduce energy consumption of buildings if working together toward a common objective (Figure 3) [21].

The basic tenet of building energy efficiency is to use less energy for heating, cooling, and lighting, without affecting the comfort of those who use the building. High-performance buildings not only save energy costs and natural resources but also mean a higher-quality indoor environment. The benefits of building energy efficiency include: reduced resource consumption, minimized life-cycle costs, reduced environmental impact, healthier indoor environment, and increased employee productivity.

One of the most critical points of biomimicry is the efficiency of resources. Given this step, four principles follow, namely: (i) multifunctional design (meet multiple needs with one elegant solution), (ii) using low energy processes (minimize energy consumption by reducing requisite temperatures, pressures, or time for reactions), (iii) recycling all materials (keep all materials in a closed loop), and (iv) fitting form to function (select shape or pattern based on need) [4].

**Figure 4:**
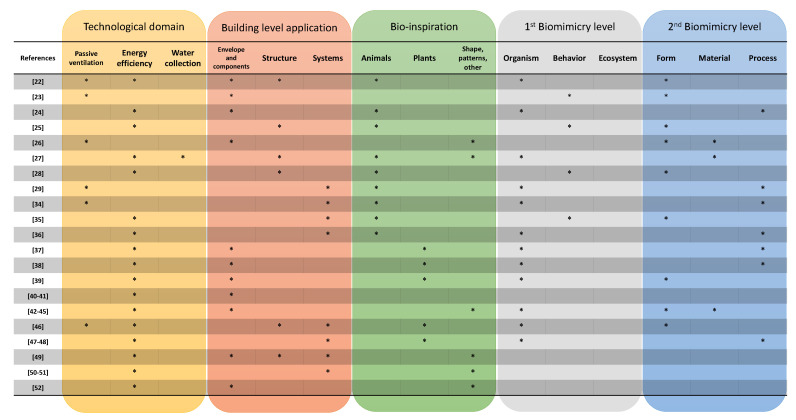
Summary of biomimicry strategies for building applications (asterisks (*) represents belonging).

Therefore, a brief literature review is presented here by filtering existing studies regarding biomimicry approaches applied explicitly to buildings for the following aspects: (i) air ventilation or conditioning systems, (ii) energy efficiency, and (iii) the minimization of resources through changes in shape or patterns. However, the approaches presented here are organized in three building-based levels: (a) envelope, (b) structure, and (c) system by differentiating the organism-based design, grouping them as animals or plants. The biomimicry approaches identified are then categorized, based on the framework presented in Table 1, by building application, biomimicry level, and technological domain (Figure 4).

### 2.1. Animals and Buildings

Depending on the location and season, emphasis is given to either cooling or heating of indoor spaces, to counterbalance the unfavorable outdoor conditions and achieve indoor comfort by controlling the indoor temperature, humidity, light availability, and air quality. The most significant advance has been in the change from rather artful design practices of indoor spaces to the specific analytical methods that are necessary to handle complex building structures.

(a) Approaches Applied at Envelope Level

The Gherkin Tower’s design, in London, was inspired by a sea sponge, *Euplectella aspergillum*, known as the Venus’ flower basket. This animal lives at the bottom of the sea, and its hexagonal skin, exoskeleton, and rounded body help it disperse the physical stress produced by strong currents. This building is essentially an elongated, curved, shaft with a rounded end that is reminiscent of a stretched egg. It is covered uniformly around the outside with glass panels and is rounded off at the corners. It has a lens-like dome at the top that serves as a type of observation deck. Open shafts built in between each floor act as ventilation for the building, and they require no energy for use. The shafts pull warm air out of the building during the summer and use passive heat from the sun to bring heat into the building during the winter. These open shafts also allow available sunlight to penetrate deep into the building to cut down on light costs. It has been said that 30 St. Mary Axe uses only half of the energy that a similarly sized tower would use [22].

The microscale pore network of the outer walls of termite nests, *Trinervitermes geminatus*, was found to improves permeability, CO_2_ diffusion, and thermal insulation. In rainy events, the micropore network allows rapid drainage of water and effective recovery of ventilation, which protects the stability of the wet nest [23].

Taking the tuna as a reference, its strategy for heat generation in the muscles organs and tissues of the inner area of its body (known as dark muscles), can be followed in the design of the floor of the office workspace to achieve a lower energy demand. Such is the case of closed spaces in buildings with high occupancy, which were shown to have lower heating demand. This lower demand implies energy savings, an energy-saving that is much greater the colder the climate of the place where the building is [24].

(b) Approaches Applied at Structure Level

Harare’s Eastgate Centre, which opened in 1996, deservedly stands as an iconic biomimetic building. Mick Pearce, the project’s lead architect, wanted the building to reflect two tenets of his philosophy of “tropical architecture”—first, that design principles developed in the temperate northern hemisphere are ill-suited to tropical climes like Zimbabwe’s; and second, that effective design should draw inspiration from local nature, which is rife with plants and animals that live there comfortably [25]. The famous building that is now a commercial center in Zimbabwe was designed, taking as inspiration the termite mounds. Their structure responds to external air movements and humidity to keep the interior fresh. However, the result shows 90% reduction of energy required for air-conditioning compared to a building of the same size. Remarkably, these designs are still based upon an erroneous conception of how termite mounds work. If we could arise from this better understanding of the structure and function of termite mounds, we could create a new outline for the biomimetic design concept in the future [2]. Termites build their mounds using zero-waste construction methods, employing a solar-powered air conditioning and developing a sustainable agriculture system. The mound created by compass termites has the shape of a flattened almond with a long north-south axis that catches the light during the day and releases heat during the night. Termites can open and close a series of heating and cooling vents throughout the walls of the mound during the day. Hence, when the interior temperature becomes too hot, vents can be opened; thus, rising warm air by stack effect [26].

Taichung Metropolitan Opera House was designed to reveal a sustainable concept in three levels. The first level is rainwater, where rainwater will be collected and filtered and reused for landscape issues, while the gray water will be reused in toilets. This opera house is designed to use recycled materials such as steel and concrete in the case the building needs maintenance or reconstruction. The architect tried to reduce carbon emissions to save the environment and reduce global warming while making people aware of the importance of sustainable materials. The form of the opera house has a smooth surface not only for aesthetic purposes but also for getting the most efficient structure, where the shell structure is the best choice because the loads are distributed equally on every part of the structure [27].

The Bird Nest Stadium took this name because the iron bars are like a bird’s nest. It was designed by simulating bird shelters, which consist of organic material such as branches and grass. The Stadium structure is innovated on the grounds of structural systems and the ways of distributing loads. Designers used the simulation technique to simulate temperatures, wind power, and humidity inside the structure similarly to birds’ nests, and to allow the audience to enjoy light [28].

(c) Approaches Applied at System Level

The heat recovery strategy of tuna’s dark muscles can be implemented to the development of an evolved design of a heat recovery ventilator with multiple cores and airflows [29]. As stated by the authors, this before provided another point of view to confront low energy consumption building designs. Moreover, the heat dissipation strategies implemented by some pinnacles for cooling by using dry surfaces (i.e., elephant’s pinna [30,31] or toco toucan’s bill [32,33]) instead of evaporation (as in humans), was applied in the development of a integrated system in buildings [34]. This integrated system was experimentally tested with the construction of a cooling panel, and by performing a further assessment, the authors presented some examples of suitable existing building facades where such heat dissipation system could be integrated.

Other strategies such as heat shields or stigmergy encountered in bees in beehives (social insects) to successfully maintain nest temperature have been evaluated to improve an integrated Peltier HVAC system for building envelopes [35]. A control strategy was theorized as instead of having an HVAC centralized control, the control of the HVAC system would be independent for each space with interconnected signals of comfort and external factors between adjacent rooms.

Moreover, bio-inspired photovoltaic applications by the properties of the fovea centralis have been investigated by [36]. They developed a light-trapping technique based on a vertical light-funnel (LF, a light-trapping scheme) silicon array, enhancing the absorption LF array about 65% in comparison with a thin continuous silicon film of the same thickness.

### 2.2. Plants and Buildings

(a) Approaches Applied at Envelope Level

The BIQ Hamburg building is covered with bio-reactive blinds that enclose the algae. These grilles allow the algae to survive and grow faster than they would otherwise while providing shade for the interior of the building. Presented in a pilot project at the International Building Exhibition (IBA) in Hamburg in 2013, the world’s first bioreactive facade generates renewable energy from algae biomass and solar thermal heat. The biomass and heat generated by the facade are transported by a closed-circuit system to the building’s energy management center, where the biomass is harvested by flotation and heat by a heat exchanger. Because the system is fully integrated with building services, excess heat from photobioreactors can be used to help supply hot water or heat the building, or be stored for later use [37]. The application of algae bioreactor as a sun screening device in building facade has not been studied extensively in terms of its solar heat transmittance or luminance. Architectural design-based research on the use and role of algae as part of the building facade system is currently being undertaken at the School of Architecture, Planning, and Policy Development in Institut Teknologi Bandung [38].

An existing, glazed 20-story office building in Pakistan was analyzed by numerical valuation, where the design of an adaptative biomimetic facade serves as a practical solution for enhancing energy efficiency without decreasing the visual comfort. The inspiration was taken from the *Oxalis oregana* leaf and mimics its abilities to track the sun path and changing its angle/position accordingly. With this designed facade, the building’s existing energy load can be decreased by up to 32% [39].

The authors in [40] investigated the ability to reduce energy consumption by applying the biomimicry approach to buildings skin design, concluding with guidelines for building skin design for more efficient energy consumption in buildings. Moreover, considering the problem of increasing cooling loads in hot climates, two buildings were evaluated in Egypt to showing the benefits of promoting biomimicry [41].

Furthermore, most outcomes of existing biomimetic approaches for designing building facades, are commonly mono-functional facades and usually with shape morphing shading devices [42,43,44,45]. This before might be a result of a lack of clarification of multi-functionality in nature from non-biologists‘ perspectives [44]. Based on the above, the authors proposed a framework for the development of biomimetic adaptive multi-functional facades. The proposed framework consisted of the following steps: (1) definition of boundary conditions, (2) selection and mapping of biological models, and (3) design generation of biomimetic facades. In which, as suggested by such a framework, the key to achieving multi-functionality in biomimetic adaptive facades lies in extensive investigation and mapping of multi-functionality in biological adaptations.

(b) Approaches Applied at the Structure Level

The building Lotus Center stands out among the common buildings of the city in the middle of the water, stimulating urban development. It houses office parts, as well as exhibition halls, meeting rooms, and conference centers. The Lotus Centre and People’s Park has become one of the most famous landmarks in Wujin with a sustained contribution to the social and cultural life of the city [46]. The project has been designed to minimize energy usage—with over 2500 geothermal piles driven through the base of the artificial lake. The entire lake water mass and the ground beneath is utilized to pre-cool (summer) and pre-warm (winter) the air conditioning systems for both the lotus and the two-story building beneath the lake. The project is also mixed mode and naturally ventilated and utilizes evaporative cooling from the lake surface to drive a thermal chimney within the main flower pod.

(c) Approaches Applied at System Level

The bionic realization of plant systems based on the recent researches on photosynthesis could be an inspiration to understand current solar utilization methodologies as the building integrated photovoltaic (BIPV) [47,48]. The authors in [47] presented a framework to integrate the photosynthesis strategy of plants to enhance energy conversion strategies as the building integrated photovoltaic (BIPV).

### 2.3. Shapes, Patterns and Others

The Beijing National Aquatic Center, located in China, is a building whose shape is inspired by soap bubbles, and which, after obtaining the most important contract, Beijing National Aquatic Center for the Olympic Games, and finishing that season, became a recreation center open to the public. It is a building whose surfaces are covered with translucent pads that are resistant to the degradation of ultraviolet light and air pollution. It contains solar panels on its roof, preserving 20% of the light that is reflected on the construction, also, solar energy is used to heat the air around the pools, and also heats the water. It can control the self-generated energy stored between the two layers of ethylene tetrafluoride ethylene by using several openings of vertical cylinders, coated upper and lower with circular panels. In winter, it keeps these openings closed, while in summer they open at low and high levels to reveal thermal energy. The surface’s material is very thin and can be cut with a knife. Still, to repair these pads, it is enough to gather the surface where it has been damaged, and it will repair itself thanks to its self-repair property while it’s under high pressure, although that will disappear over time [49].

Lunt and colleagues at Michigan State University pioneered the development of a transparent luminescent solar concentrator that when placed on a window creates solar energy without disrupting the view. The thin, plastic-like material can be used on buildings, car windows, cell phones, or other devices with a clear surface [50]. Transparent and semitransparent power-producing sur-faces have been developed for improving the utilization of solar energy without disrupting the aesthetic of surfaces. For example, transparent photovoltaics have been fabricated by exploiting the excitonic character of organic semiconductors, which selectively absorb near-infrared light and have reached efficiencies in the range of 2–4% for high transparencies. The near-infrared transparent luminescent solar concentrators based on organic salts provide an alternative strategy for transparent solar harvesting systems that can ultimately enhance the overall system efficiency of combined UV and near-infrared transparent luminescent solar concentrators [51].

Council House 2 (CH2) is a significant example of an ecologically sustainable development building. The CH2 building incorporates many innovative sustainable technologies in its design. It includes phase-change materials for cooling, undulating high thermal mass concrete ceilings for passive radiant cooling, photovoltaic cells powering a facade of louvers, automatic night-purge windows, solar shading, shower towers for low energy cooling, rooftop solar collection for water heating, green roofscape, and glare control. A vital feature of the building is that it provides 100% fresh air, with an air change every half hour. This benefits the occupants in terms of health by providing superior indoor air quality and conservation of energy costs. It has been estimated that the savings made by using this system will pay for the building within 5 to 10 years. Other elements that have been incorporated into the design include recycled concrete, recycled timber, timber windows, sewer mining, and co-generating using natural gas. Shower towers and phase change material have been employed to produce and store cold water for use by chilled ceilings and beams, while wind turbines are used to extract air during a ‘night purge.’ Solar hot water heating and photovoltaics were implemented, taking advantage of good solar access as a result of CH2’s [52].

## 3. Methodology

**Figure 5:**
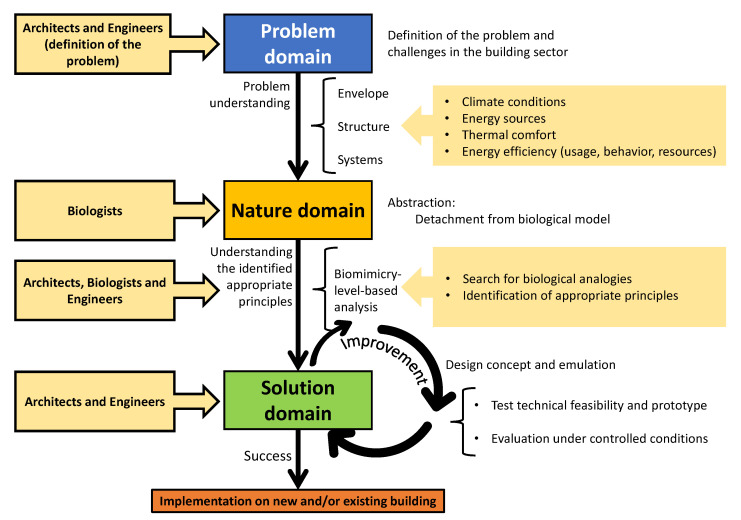
Proposal for choosing a problem-based approach.

In order to define a framework, the methodology adopted here, is based on the problem-based approach (Figure 5) [8]. Thus, the investigation presented here starts by defining the problem in need of a solution. Such problems, as stated in Section 1.1 and Section 1.2 lies in the local energy goals which struggle with climate conditions and look for solutions. The proposed methodology consists of the following major points:

- Definition and understanding of identified problems.
- Definition of the case study.
- A comprehensive literature review of biological analogies, based on the problem understanding.
- The classification and categorization of such identified biological analogies and biomimicry strategies, depending on a possible application at a specific building level.

The main biomimicry-based strategies interested here are passives, which can solve problems by triggering either the envelope level or structure level. This before is based on the hypothesis that nature already addresses must of our indoor-environment-related problems, which solutions may lay on specific or a combination of pinnacles’ strategies and principles.

**Figure 6:**
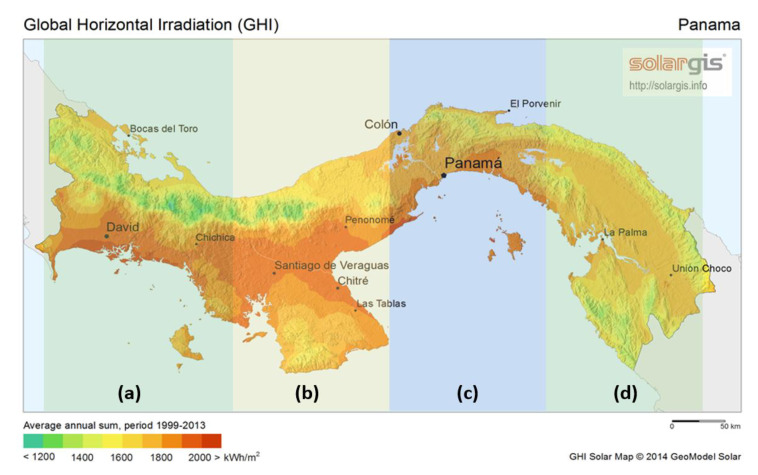
Areas with different climate-types within the humid tropical climate in Panama: (**a**) area with tropical forest and template-like zone, (**b**) arid area, (**c**) highly urban regions rounded by tropical forest, (**d**) area with heavy tropical forest. All areas possess coastal zones. SolarGIS © 2014 GeoModel Solar.

To define the case study for our theoretical framework, climate-related problems were targeted and analyzed for each of the three most encountered types of climates in Panama (Figure 6). By understanding the identified climate-related problems, such problems, due to heat, air, water, and light as defined in [43], can be categorized as having a strong link to a specific or a combination of building-based level applications: envelope, structure or systems. Identifying such a link might be crucial in leading to successfully filtering available biomimicry-based concepts along with biological analogies found in different specific climate-related pinnacles, which leads to ease of the spent-time in the concept-design and emulation phase. In this manner, the case study conducted here is limited to better understand both transitions between the problem domain to the nature domain, and from the latter to the solution domain. Nevertheless, the transition from the solution domain, based on concept-design and emulation success, is intended to be assessed by performing a SWOT analysis in the discussion section.

## 4. Implementation of the Proposed Methodology and Results

Panama is a part of Central America. It is bordered by Costa Rica (west), Colombia (east) and the Pacific, and Atlantic Oceans (north and south). Geographically recognized as a country with a strategic location because it is the connection between North and South America; therefore is a hub for any connection through land, sea, or air. Panama knows as the Hub of the Americas. The capital is Panama City. The total population has been increasing in the last few decades. By 2017, 65% (2.7 million) of the total population lived in the capital [18].

Being a country with mostly tropical climate as shown in Figure 6, several types of biomimetic strategies investigated and presented before can be applied to buildings for energy efficiency, by covering the facades to take advantage of the heat in the dry season or get water from torrential rains in the rainy season. Panama City is starting with the use of solar energy, but most of the last buildings’ structures have entire glazed facades. Thus, if we imagine a strategy such as [50] fully developed, where all buildings in the future will use these transparent solar panels, the entire city could generate energy and share clean energy to the surrounding communities, improving the quality of life of many people unimaginably.

In this matter, Table 2 summarizes the identified pinnacles and biomimicry-level-based strategies that can be applied in buildings depending on the type of climate-related problems in coastal areas, among the tropical forest, and arid areas.

### 4.1. Buildings in Coastal Areas

(a) Problem Definition

Some cities are built near the coast, things that costs a lot because of the amount of erosion in the pillars that hold the structures or ground near the building and salt in the air mixed with constant winds. Due to the salinity, rust is a reaction that presents itself mostly near the coasts, which is the case for Panama (Figure 6). To find a suitable solution for this problem, we relied on the study of biological microstructure, which is one of the most critical research areas in biomimicry. In this case, the most suitable biomimicry-level strategies may lie in the organism material, and the building-level application lies in the envelope level.

(b) Identification of Biological Analogies and Principles

**Figure 7:**
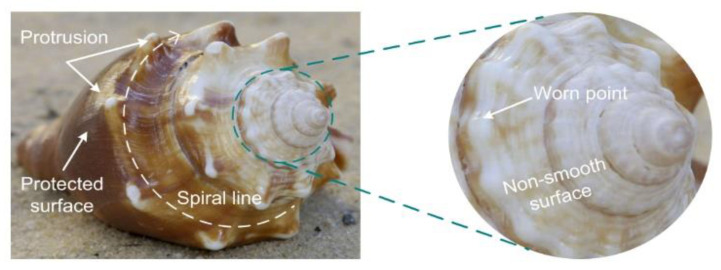
Biological characteristics of a conch [53].

The waves crash constantly? Conchs suffered the same until they adapted to the surf. The conch is generally fusiform, and the middle shoulder is raised. The overall screw shell presents a non-smooth surface. A spiral emits from the tail and extends to the shoulder. There are prominent protrusions on the spiral line (Figure 7), where several turns of ribs and small nodules, are among the spirals that give the conical outer shell a non-smooth surface. Comprehensive analysis shows that these protrusions, spirals, and non-smooth surface morphology can reduce the erosion of sediment. The outer shell of the conch is well protected against abrasion and erosion [53].

**Figure 8:**
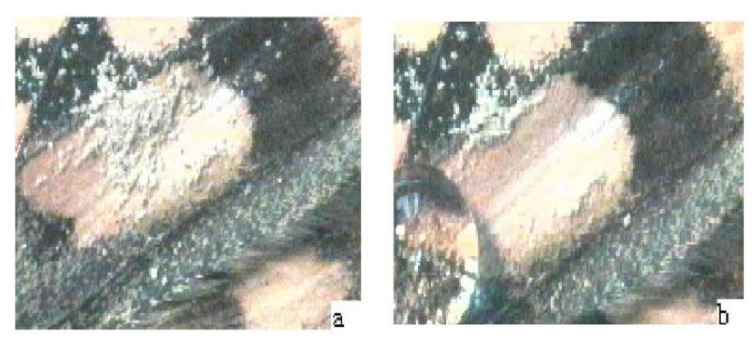
Photograph by the confocal light microscope. (**a**) Some dust on the wing surface. (**b**) The moving spherical water droplet can clean the dust on the wings surface [54].

Similarly, buildings’ structures sometimes get damaged by the salt in the air. By applying an effect like butterflies’ wings, the salt can slide off when it gets wet. After the butterfly emerges from its chrysalis, the wings cannot grow or change anymore. They also cannot self-repair if the wings are damaged or destroyed. To reduce the influence of wind, rain, fog, dew, and dust, the butterfly wings’ surface has evolved to have water repellent and self-cleaning capabilities. Most butterfly wings surfaces are smooth at the macroscopic view (Figure 8), except that somebody’s hair can be observed. However, with the help of the microscope, we can find that the surfaces are composed of squamous (a non-smooth surface), like the conch. This kind of surface has unique characteristics, lotus-effect, where self-cleaning is one of them [54].

### 4.2. Buildings Surrounded by Tropical Forest

(a) Problem Definition

In buildings surrounded by forests or places with high humidity, the structure tends to deteriorate fast, and the people inside tends to fall in discomfort, thanks to the constant change of temperature, which leads to acquiring air conditioners. So, if we search for sustainable solutions, we must look for our surroundings in nature.

In this case, the problem understanding helps realized the different building level applications that can be implemented to give solutions for such humidity-related problems. Regardless, such problems can be tackle by applications either implemented at envelope level, structure level, and system level. Rather well-established solutions have been implemented in tropical regions even before such knowledge was formally defined in the literature. Such knowledge, far from being related to any pinnacle strategy, is based on bioclimatic architecture definitions [58,59,60,61,62].

(b) Identification of Biological Analogies and Principles

Like the nest of social wasps, it is demonstrated that moisture and latent heat significantly influence the thermal performance of the nest construction. Two colonies of the hornet *Vespa crabro* were investigated to clarify the relationship between the temperature and the moisture regime inside the nest. For fairly stable nest temperatures, the hornets maintain a high relative humidity inside the nest. Researchers found that, as a consequence, a partial vapor pressure gradient between nest and ambient, drives a constant vapor flux through its envelope. The vapor flux is limited by the diffusion resistance of the envelope. The driving force of vapor flux is heat, which is consumed through evaporation inside the nest [55]. Such a strategy applied to buildings envelopes can be challenging regarding design, envelope internal deterioration, and maintenance.

**Figure 9:**
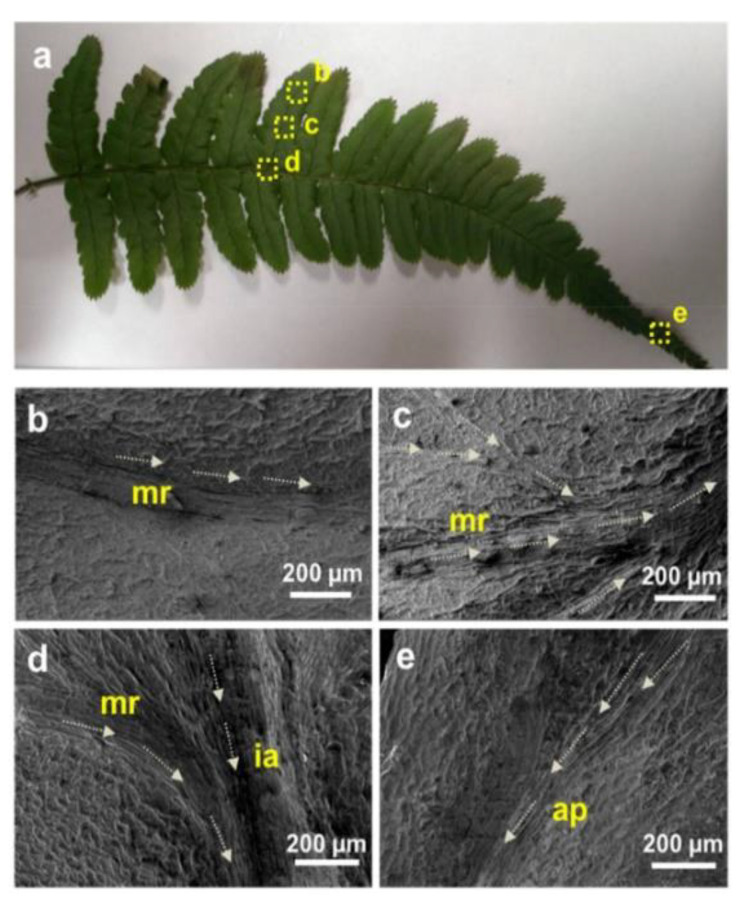
(**a**) Photograph of the *D. marginata* frond with labeled areas showing different locations of the water channels. (**b**–**e**) Scanning electron microscopy micrographs of the labeled areas in (**a**); arrows indicate the presumed direction of the water transport through these channels (ap: apex; ia: inner axis; mr: mid-rib) [56].

On the other hand, the fog harvesting properties of *Dryopteris marginata*, which surfaces have a remarkable ability to channel water rapidly and efficiently. This can be attributed to the integrated system of the multiscale channels and surface microstructures. The surface of the real leaf was replicated using a facile soft lithography technique and good channeling properties (Figure 9). The replicated surfaces proved that efficient water-channeling is due to the surface microstructures rather than the surface chemical composition. This understanding of efficient and well-directed water transport and collection due to the intercalar network of the microchannels in the *D. marginata* leaf provides a promising approach to design efficient surfaces for application [56]. Building facades with such a strategy might not only be useful in humid areas for drinkable water collection, but they might also be helpful in arid areas where water is limited.

### 4.3. Building in Arid Areas

(a) Problem Definition

In these areas (Figure 6), due to excessive solar radiation during daytime with no available shadowing from nearby taller buildings, the building roof suffers the most, mainly because of no high-cloudiness skies due to low levels of water content in surrounding air and soil. Even though various bioclimatic strategies exist to overcome envelope overheating [63,64], occupants still experience sweatiness due to surrounding hot air. During the nighttime, natural ventilation might not be suitable despite the temperature drop; for these areas, the day-night temperature variation is not significant. Buildings with envelopes capable of changing its radiative properties or withdrawing water from fog might be of a further assistant.

(b) Identification of Biological Analogies and Principles

To optimize our buildings in these areas, deserts have a good number of land fauna, consisting primarily of foxes, camels, small rodents, and reptiles [65]. In general, these animals can alter their internal environment and possess numerous adaptative strategies, to respond to this kind of climate. Certain insects and plants have evolved to have superhydrophobic and superhydrophilic domains patterned on a surface, in order to collect water from the surrounding environment efficiently. Examples of this are the Namib desert beetle, the cactus Opuntia microdasys from the Chihuahuan desert, and the Sand Lizard. They can collect water from fog, due to their spines and their surface morphology. The spines contain microgrooves with a higher roughness near the tip than near the base, creating a wettability gradient along the length of these features, drawing water towards the cactus’ skin. Besides, coloration is an important factor in reducing heat absorption; thus, a lighter colored coat is more familiar in desert animals.

Moreover, insects, to control their wettability, they must simultaneously take advantage of two parameters: their surface energy and their surface morphology. Recent research based on these beetles has demonstrated an interesting connection between water and the insect cuticle surface. For example, color-switching has been observed in the Dynastes genus, depending on the air humidity. With the awareness that a closer investigation of giant beetles can inspire new materials, where a remarkable example of giant beetles is the Goliathus genus. Regardless of the external environmental conditions, the Goliathus specimen always presents an appearance with distinct white markings. An interesting aspect of these white parts is that they remain clear and clean. Significant differences are in the surface properties between the black and the white parts. While the black parts are slightly hydrophobic, the white parts are highly hydrophobic with strong water adhesion (parahydrophobic character), similar to what is observed on rose petals.

**Figure 10:**
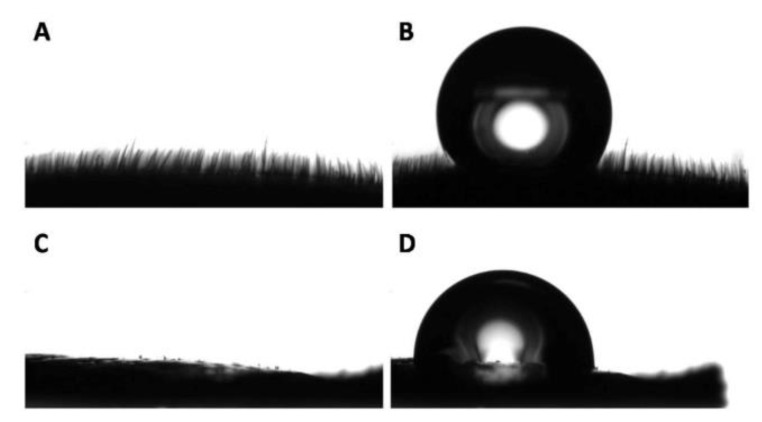
Representative side view of the white and black surfaces. (**A**) Profile of the white part of a *G. orientalis* elytron and (**B**) water droplet deposited on its surface. (**C**) Profile of the black part of a *G. orientalis* elytron and (**D**) water droplet deposited on its surface [55].

The side view of the white surface was observed with the goniometer camera, revealing the presence of microscopic hairs that covered the entirety of the surface (Figure 10A). The presence of microhairs on the white part made the observation and discernment of the triple point complicated, precluding the accurate analysis of the contact angle (Figure 10B). Interestingly, the side observation of the black part of the elytron did not reveal hair-like microstructures (Figure 10C), and the apparent contact angle of the black part was measured with more accuracy (Figure 10D) [55].

The cactus, *Opuntia microdasys*, in desert possesses a continuous and efficient fog collection system originating from the cooperation of Laplace pressure gradient and the wettability difference. Recent research has revealed that geometrically conical-shape spines and hierarchically hydrophilic/hydrophobic dichotomy of the cactus cluster play an important role in the fog collection. Firstly, driven by Laplace pressure difference, the water droplet could directionally move towards the base of the hydrophobic conical spines. Subsequently, the hydrophilic trichomes, a hair-like fiber cluster grown at the base of the spines, could rapidly absorb and transport the collected water into the stem of cactus [57]. This before can lead to self-cleaning facades with low maintenance in arid areas, and also, to reduce lack of water for household usages, by implementing innovative morphologies for the building skin.

## 5. Discussion: Towards the Solution Domain

**Figure 11:**
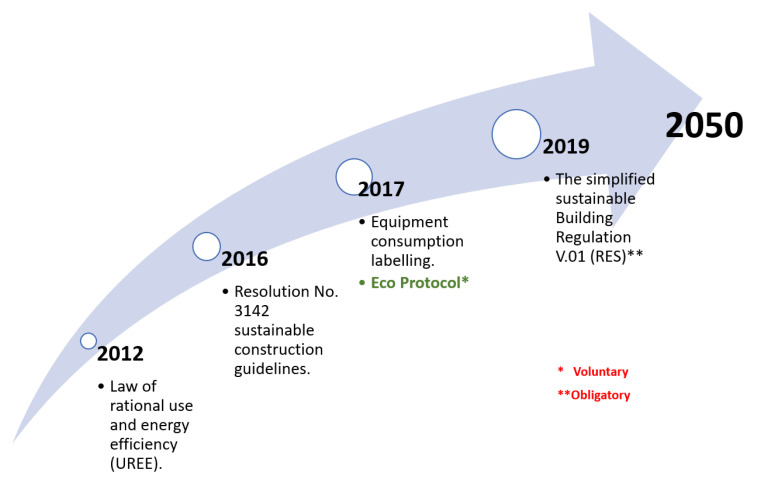
Chronologic timeline for recent regulations establishment in Panama.

As presented before in Section 1.1, the motivations of this investigation are to bring options to the increasing demand for reducing the energy consumption in the building sector. Since 2012, Panama has been increasing their effort towards the rational energy use and energy efficiency in buildings (see Figure 11), through the inclusion of the Guidelines for Sustainable Construction (Resolution No. 3142) in 2016. Moreover, in 2017, the implementation of energy consumption labels for building equipment was included, followed by the development of an “Eco Protocol” which give recommendations of the energy and water usage, for reducing consumption in existing buildings (retrofitting) [66]. Such recommendations are based on the climate analogy between Panama and Singapore, which has a tropical rainforest climate with relatively uniform temperature and pressure, high humidity, and abundant rainfall.

However, at this point, all regulations have been only voluntary. In contrast, in 2019, Panama incorporates an obligatory regulation concerning the characteristics of new buildings designs and construction, by focusing on the envelope materials, depending on the building type, and the air conditioners’ COP values [67]. These regulations assure a 20% reduction in energy consumption concerning the current construction approaches. By 2034, it is expected that 100% of the population will have access to electricity [18]. Finally, by 2050, as established by the National Energy Plan together with the National Institute of Statistics and Census (INEC in Spanish), population growth of 46.1% (5,625,442 people) is expected with respect to 2013 [18].

As presented in [18], among the regulations mentioned before, it is stated that a measure to be taken is the restriction applied to the most consumable equipment (air conditioning, lighting, refrigerator, and television), in terms of energy efficiency indicators, based on the equipment labeling regulation. It is estimated that the implementation of these regulations, with respect to a reference scenario, can reduce the energy consumption in only 30% for new constructions, and a reduction of only 7% for existing buildings through retrofitting strategies established in [66].

To contribute to the efforts to further reducing the energy consumption of existing and new buildings to meet the goals by 2050, the introduction of biomimicry strategies for the retrofitting of existing buildings and new buildings designs shows promising opportunities. To evaluate the potential of such strategies in Panama, a SWOT analysis was performed (see Table 3). SWOT analysis is applicable to concepts, as well as company or market evaluations. Strengths and weaknesses are to measure the existing internal environment, while opportunities and threats are for the external environment [68,69]. It is a fundamental method to implement when initiating new concepts in the industry.

The SWOT analysis performed here is inspired by the analysis presented in [70] and adapted to the context in Panama. The internal elements, corresponding to the strengths and weaknesses, can be easily addressed by focusing on the availability of resources such as personal assets, consumers’ perception, complexity, among others.

For the strengths of implementing biomimicry strategies in buildings designs, the most important would be to meet the future energy local regulation demands due to higher effectiveness in the energy efficiency of buildings by implementing biomimicry-based strategies for envelope improvements. This higher effectiveness can cause higher prestige levels, as well as creating more climate adaptability of cities while assuring comfort limits for indoor and outdoor environments. Besides, biomimicry-based-designed buildings could be considered as of higher value due to more environmental-friendly feeling, comfort reliability, which also could increase the rental costs and raise the local economic potential.

Finally, the need for multidisciplinary collaboration to accomplish biomimicry-based-designed buildings brings an increment in the competitivity regarding more trained human-assets regarding 3D modeling and widening the standard-construction-sector thinking. However, the biomimicry-based-designed buildings may include some weak aspects, such as the lack of systems expertise, with respect to the higher dependency on software-related design arising from the need for 3D modeling. This before provokes the need for a multidisciplinary workforce, which from the point of view of today’s availability of trained professional assets, weakened a robust starting adaptability and acceptability of implementing such strategies.

On the contrary, this lack of systems expertise can also be seen as an opportunity, since education plays an important role, biomimicry-based thinking or design would be needed to be included in the architecture and other disciplines syllabus. Similarly, the need for multidisciplinary workforce might bring collaboration with experts in tropical pinnacles, such as the researchers and database at The Smithsonian Tropical Research Institute [71]. This would certainly establish solid basis to support the conceptualization of biological analogies, and help the transition from the nature domain to the solution domain (Figure 5). Another paradigm-shift hold-back may be due to the lack of interest from the construction sector, due to the increment in design complexity, which also may influence higher construction costs.

In actuality, the energy consumption in the building sector is firstly related to the conditioning of indoor spaces, mostly cooling and followed by lighting. In this matter, different nature strategies can be mimic to strongly impact cooling and lighting, such as the ones presented before in Section 2, which is based on the analysis presented in Section 3. A methodology for deciding which biomimicry-level-based strategies to apply, can be generalized, following the framework presented earlier in Table 1 combined with Figure 1, which resulted in the schematic presented in Figure 5).

Moreover, one of the most crucial hold-backs to the introduction of such biomimicry-based thinking might be the lack of mechanisms for monitoring compliance with regulations. Currently, there is no laboratory to certify compliance with equipment labeling, which can be extrapolated to the rest of the measures and regulations that are being implemented recently and resulting in a lack of information regarding the real energy consumption of buildings.

Furthermore, the introduction of biomimicry strategies in buildings designs is a multidisciplinary task, which can be included in BIM techniques to get better results, as well as to provide additional knowledge, requiring the interaction of different specialists in the natural sciences and engineering. This could bring the development of new ideas for passive strategies for reducing energy consumption in buildings, which can provide sustainability as in nature.

## 6. Conclusions

A comprehensive analysis of available research regarding biomimicry-based approaches destined to improve buildings’ designs, has been presented here, driven by the increasing energy regulations demands to meet future local goals and with the objective to propose a framework for applications in Panama. Such biomimicry-based approaches have been further analyzed and evaluated to propose the incorporation of organism-based design for three of the most climate types found in Panama.

Consequently, a SWOT analysis helped realized the potential that biomimicry-based approaches might have in improving the odds of meeting the demands of the local and global regulations, based on the ODS and the COP21 Paris agreement. In the Panama context, the energy consumption in the building sector is mostly related to the conditioning of indoor spaces, mostly cooling and lighting. Different nature strategies can be mimicked to strongly impact these two aspects in the building sector, such as the ones presented here. Regarding the strengths of implementing biomimicry strategies in building designs, the most important would be to meet the future energy local regulation demands in buildings by implementing biomimicry-based strategies for envelope improvements. The need for multidisciplinary collaboration to accomplish biomimicry-based building designs, brings an increment in the competitivity regarding more trained human-assets, widening the standard-construction-sector thinking. Finally, the analysis presented here can serve as the foundation for further technical assessment, via numerical and experimental means.

## Figures and Tables

**Table 1:** A framework for the application of biomimicry (adapted from [10]).

Level of Biomimicry		Example—Case of a Building That Mimics Termites:
Organism level (Mimicry of a specific organism)	Form	The building looks like a termite.
	Material	The building is made from the same material as a termite; a material that mimics termite exoskeleton/skin for example.
	Construction	The building is made in the same way as a termite; it goes through various growth cycles for example.
	Process	The building works in the same way as an individual termite; it produces hydrogen efficiently through meta-genomics for example.
	Function	The building functions like a termite in a larger context; it recycles cellulose waste and creates soil for example.
Behavior level (Mimicry of how an organism behaves or relates to its larger context)	Form	The building looks like it was made by a termite; a replica of a termite mound for example.
	Material	The building is made from the same materials that a termite builds with; using digested fine soil as the primary material for example.
	Construction	The building is made in the same way that a termite would build in; piling earth in certain places at certain times for example.
	Process	The building works in the same way as a termite mound would; by careful orientation, shape, materials selection and natural ventilation for example, or it mimics how termites work together.
	Function	The building functions in the same way that it would if made by termites; internal conditions are regulated to be optimal and thermally stable for example. It may also function in the same way that a termite mound does in a larger context.
Ecosystem level (Mimicry of an ecosystem)	Form	The building looks like an ecosystem (a termite would live in).
	Material	The building is made from the same kind of materials that (a termite) ecosystem is made of; it uses naturally occurring common compounds, and water as the primary chemical medium for example.
	Construction	The building is assembled in the same way as a (termite) ecosystem; principles of succession and increasing complexity over time are used for example.
	Process	The building works in the same way as a (termite) ecosystem; it captures and converts energy from the sun, and stores water for example.
	Function	The building is able to function in the same way that a (termite) ecosystem would and forms part of a complex system by utilizing the relationships between processes; it is able to participate in the hydrological, carbon, nitrogen cycles etc. in a similar way to an ecosystem for example.

**Table 2:** Summary of identified pinnacles and biomimicry-level-based strategies for given building applications.

Area Type	Challenges for Building Sector	Pinnacles’ Strategies	Biomimicry Level	Building Level Application	Technological Domain (Objective)
Coastal	Erosion, rust, air salinity, strong winds	Shells, conch: The protrusions, spirals, and non-smooth surface morphology can reduce the erosion of sediment [53].	Organism Material	Surface envelope	Self-cleaning façade
		Butterfly wings: Its surface has evolved to have water repellent and self-cleaning capabilities [54].			
Forest	Surrounding air with high humidity	Social wasps: Moisture and latent heat significantly influence the thermal performance of the nest construction [55].	Behavior Form	Structure	Facade component to channel air moisture
		Dryopteris marginata: Can channel water rapidly and efficiently [56].	Organism Function and Process	Envelope and system	
Arid	Low water content in surrounding air and soil. Day-night temperature variation is not significant	Goliath orientalus beetle: While the black parts were slightly hydrophobic, the white parts were highly hydrophobic with strong water adhesion, like what is observed on rose petals [55].	Organism Function and Process	Envelope and system	Water harvesting and conservation
		Cactus: Have a continuous and efficient fog collection system [57].			

**Table 3:** SWOT analysis applied for the introduction of biomimicry approaches to building designs (based on [70], and adapted to our context in Panama).

SWOT Analysis	Strengths	Weaknesses
Internal	Higher effectiveness in the use of energy, thus, the energy consumption remains within the local regulation limits. Higher prestige level. Climate adaptability. Comfort improvement. Higher value and rental costs. Increases competitivity regarding higher 3D modeling dependency, thus, the need of training human resources and collaborationincreases.	Lack of mechanisms for monitoring compliance with regulations. Paradigm shift for the construction sector: design and structural. Higher dependency on 3D software-relateddesign management. Lack of interest from the construction sector, due to an increment in complexity design. Higher design cost values, due to the increment in complexity design.
	**Opportunities**	**Threats**
External	Response to the increasing demand of more environmentally friendly buildings. Sustainability focused development policy. Technological developments. Lack of local nanotechnology-based industries. Lack of systems expertise. The need for multidisciplinary workforce.	Materials features do not comply with standards. Difficulty in project financing. Increase in dependency on external aid for the evaluation and decision-making-process.
